# An Interesting Case of Exophthalmos

**Published:** 1873-08-15

**Authors:** F. C. Hotz

**Affiliations:** Ophthalmic Surgeon to Cook County Hospital


### AN INTERESTING CASE OF EXOPHTHALMOS.

BY DR. F. C. HOTZ, OPHTHALMIC SURGEON TO COOK COUNTY HOSPITAL.

On March 27th an Italian was admitted to the Eye Ward of the hospital. Unfamiliar with the English language, he could give us but very little information about his troubles. From his friends we occasionally learned that some months ago he received a severe blow on his left eyebrow, and during the two weeks preceding his admission to the hospital he had erysipelas of the face.

We found a large abscess of the left upper lid; the pus had broken through the external surface, but the opening was too small to allow a free escape of the matter.

Although greatly swollen, the upper lid did not hide the eyeball completely. This was of a normal size, but pushed from its natural position considerably forward, and with a slight downward and inward tendency. In this position the globe was perfectly immovable. The conjunctiva was very hyperæmic, and œdematous to such an extent that a thick, succulent fold protruded from between the eyelids, and encircled the cornea. This membrane, and the aqueous humor, were normal; the iris was adherent to the capsule of the lens by two posterior synechiæ, but of a normal color and brilliancy; the pupil was small and active. The vitreous humor was so clouded that an examination with the opthalmoscope was impossible. The eye was totally blind, being unable to discern light from shade.

The opening of the abscess in the upper lid was enlarged and the pus thoroughly emptied. By probing the abscess we could not discover either any foreign substance or necrosis, or caries of the bones; and we found the suppuration limited to the cellular tissues of the lid.

Between the supra orbital margin and the protruding eyeball, somewhat above the external canthus, the conjunction was bulging out the most, and seemed to fluctuate. A narrow-bladed scalpel was therefore pushed through the conjunctiva into the cellular tissue of the orbit, to the depth of one inch ; but no purulent matter appeared at that time, nor when the puncture was repeated two days later.

By the use of warm water dressing, the abscess of the lid was healed very soon. No sooner had the lid recovered its normal size when a diffuse swelling of the periosteum, along the supra orbital margin became apparent; a pretty solid exudation made it impossible to put the tip of the finger under that margin.

Iodide of potassa (5 grains *pro dosi*) was given internally; the tincture of iodine was rubbed into the skin over the left eyebrow; and a gentle pressure was used on the globe, by means of a bandage, due care being taken to protect the exposed cornea.

After two weeks' treatment the œdema of the conjunctiva had subsided almost entirely; the protrusion was diminished, and the eyeball slightly movable. After another week the globe had returned to its normal position and recovered its full mobility; the vitreous humor was less cloudy, and the patient could count the fingers at a distance of several feet. In this way the patient continued to improve, and when he left the hospital (May 21st) the vitreous humor was perfectly transparent; the fundus oculi did not show anything abnormal, save the outlines of the optic disc being somewhat indistinct. The sight was very satisfactory, but could not be ascertained accurately, for the reason that the patient had never learned to read. The periosteal exudation was re-absorbed, and the left supra orbital margin appeared to the touch like the right.

*Remarks.*—Our first impression of the case was that the exophthalmos was caused by an acute inflammation of the cellular tissue of the orbit, which generally terminates in an abscess. The rapid development of so great a protrusion; the immobility of the eye; the congestion and œdema of the conjunctiva; the fluctuation—all these symptoms spoke strongly for this diagnosis.

The patient having been struck on his left eye, two months previously, we could think of a foreign body lodged in the orbit, or of necrosis, as the cause of the abscess; but we found neither. So we took the inflammation in the orbit for a sequelæ of the erysipelas. It sometimes occurs in erysipelas of the face that the inflammation extends beyond the cutaneous tissues. In our case it had produced an extensive suppuration in the upper lid, and we could well expect to find a similar morbid process behind the protruding eyeball. The exploratory incision, however, disappointed our anticipation, although it was made deep enough, and at that place where all symptoms indicated the presence of pus. But, in fact, there was no pus in the orbit. The protrusion of the eye was due simply to the œdema of the cellular tissue of the orbit, caused by an extensive periostitis of the roof of that cavity. This could be found out after all swelling of the upper lid was removed. The œdema was successfully acted upon by pressure bandage (which would not have been tolerated by the patient had there been any *cellulitis*), and it disappeared entirely when the periostitis was effectually treated by iodine.

The cornea, although deprived of the protection of the lids during two weeks, escaped all damage, because it was kept covered carefully and continuously with fine oiled silk. The impairment of vision was due chiefly to the stretching of the optic nerve, but partly also to an œdema of the retina, choroid, and vitreous humor, brought about by a stasis of the circulation in these tissues. With the removal of the causes the sight quickly returned.

